# A Strategy toward Collaborative Filter Recommended Location Service for Privacy Protection

**DOI:** 10.3390/s18051522

**Published:** 2018-05-11

**Authors:** Peng Wang, Jing Yang, Jianpei Zhang

**Affiliations:** 1 College of Computer Science and Technology, Harbin Engineering University, Harbin 150000, China; wpeng68@yahoo.com (P.W.); zhangjianpei@hrbeu.edu.cn (J.Z.); 2 College of Information Engineering, Suihua University, Suihua 152000, China

**Keywords:** location services, position profile, density prioritization, collaborative filter

## Abstract

A new collaborative filtered recommendation strategy was proposed for existing privacy and security issues in location services. In this strategy, every user establishes his/her own position profiles according to their daily position data, which is preprocessed using a density clustering method. Then, density prioritization was used to choose similar user groups as service request responders and the neighboring users in the chosen groups recommended appropriate location services using a collaborative filter recommendation algorithm. The two filter algorithms based on position profile similarity and position point similarity measures were designed in the recommendation, respectively. At the same time, the homomorphic encryption method was used to transfer location data for effective protection of privacy and security. A real location dataset was applied to test the proposed strategy and the results showed that the strategy provides better location service and protects users’ privacy.

## 1. Introduction

With the rapid development of network technology and mobile terminals, location-based services (LBS) are being widely used for providing corresponding location services, such as searching for nearby shops, hospitals, tourist attractions and route navigation services and so on [1]. Under the existing LBS service architecture, position information and inquiry requests are sent to a LBS server using smartphones and other devices and the corresponding location service returned according to the position of users. From the location service architecture point of view, the existing location service generally adopts a centralized server form and hot recommendation for service results. According to distance, price, score, etc., the service results are sorted and secondarily screened by users to obtain a final result. This common location service solution has however some drawbacks: (a) due to the hot-spot recommendation, location service results are more popular and one cannot provide higher quality LBS service results that meets individual needs; (b) the location service process cannot guarantee the privacy of the users’ security issues, and may even reveal the users’ position information. Thus, protecting the position privacy of users and providing better location services has become a hot research topic nowadays.

In the relevant location service system including privacy protection, the k-anonymous privacy protection method was firstly proposed by Gruteser and Grunwald [2] in a location service [3]. A differential privacy protection method was applied in the location service field in [4]. On the other hand, Chow et al. [5] summarized the location privacy protection scheme from the aspect of system structure. Most of the existing location privacy protection schemes adopt a centralized structure with a trusted anonymous server, and the accurate positions of the users are generalized to meet the needs of the area. It is clear that the trusted anonymous server in the centralized structure could become a communication bottleneck and the key target of any attack. A spatial anonymous method based on a peer to peer structure was proposed by Chow et al. [6]. To some extent, it could reduce the shortcomings of the centralized location server. This method mainly solved the problem of using K-anonymity privacy protection in distributed systems. In their paper, the authors assumed that the nodes were trustworthy, but the privacy protection between adjacent nodes was ignored. Shokri proposed a LBS service with a neighboring users sharing mechanism to reduce the users’ exposure to the server. In their paper, the node set up a buffer to store the LBS service results obtained from the server in the past and provided location services, but privacy issues of cold-start and initial LBS service requests still exist in service [7]. In our previous work, a recommended location service was proposed using neighborhood node position information based on a distributed system structure. The previous study provided a model framework of the recommended location service, but the method had some shortcomings result in low availability of filtered proximity users’ position information and low quality service results [8]. The shortcomings were mainly reflected in the following two aspects: (a) the position data set is not preprocessed; (b) the neighbor selection mechanism is relatively simple, only considering of geographical location overlap, and the similarity matching mechanism among users is ignored. The abovementioned vulnerabilities need to be urgently solved.

In the present study, we aim to develop a new location service method. Position profiles with user preference are established using users’ position information based on a distributed structure. Then, the position data is preprocessed using a density-based clustering algorithm and neighboring users were established using a density measure algorithm. Location service recommendations were designed using two collaborative filtering recommendation algorithms based on position profiles and position points. At the same time, the privacy protection of the user position dataset was considered, and the homomorphic encryption method was used to transmit position data among neighboring users. In addition, this scheme makes full use of the characteristics of a distributed system, and distributes the computing tasks of recommended services to neighboring nodes, which effectively solves the problem of overload of key nodes in existing schemes. Above all, our main contributions can be summarized as follows:

1. This paper proposes a method to construct user position information profiles based on density measurements. The collaborative filtering recommendation method was used to recommend location services to reduce the frequency of server access, avoid privacy leaks and improve the service quality.
2. According to the characteristics of the location service system, two different collaborative filtering recommendation methods based on position profile and position point were designed, respectively. Two different similarity measures were adopted. The Paillier cryptosystem was applied to the users’ position profiles.
3. The feasibility and validity of the algorithm were verified on a real dataset. Some factors including data utility and communication cost were compared with the existing methods.

The article is organized as follows: the related work is described in the Section 2. The work process of collaborative filtering recommended location service strategy is demonstrated in the Section 3. The security and characteristics of the proposed strategy are analyzed in Section 4. The feasibility and performance of the proposed algorithm are verified in Section 5. Conclusions and future perspectives are provided in Section 6.

## 2. Related Work

**Figure 1:**
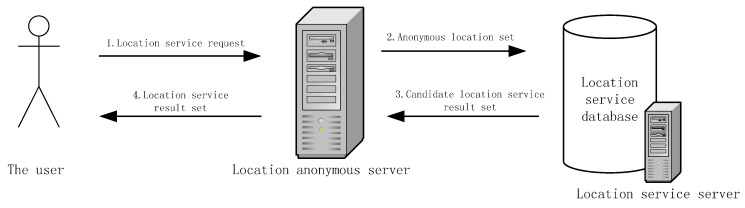
The diagram of centralized location service.

Most of the existing location-based service architectures generally adopt a centralized anonymous location service including a three-tier model. The steps include: (1) users send location service requests and their positions to the location anonymous server, (2) K-anonymization is performed on the received positions by the location anonymous server and then the data is sent to the location server, (3) the location server returns the location service candidate set to the location anonymous server, (4) the service result is sent to the users by the location anonymous server. The corresponding architecture diagram is shown in Figure 1.

**Figure 2:**
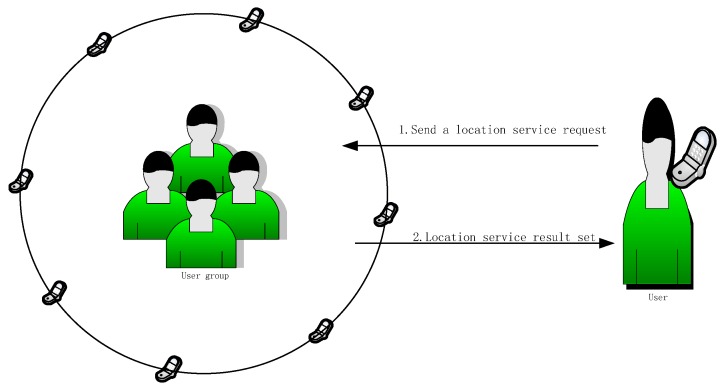
The diagram of a distributed location service.

Compared with the centralized anonymous location service architecture, there are other architectures based on distributed location services. For example, reference [6] provided a k-anonymity method with a peer-to-peer system. Reference [7] provided a distributed structure with caching mechanisms for location services. In [8] location services based on a distributed architecture were recommended. When a user needs a location service, the location service request is broadcast to a group of neighboring users, and a neighboring user who meets the measurement conditions provides a location service result using its own location information. The process is shown in Figure 2.

The two location service systems were compared and analyzed. The centralized location service system is widely used and provides more popular location service results. However, there are obvious disadvantages in this system: (1) the centralized anonymous location server and location server would become communication bottlenecks and attack points. Once the location server fails to provide location services and the servers have been attacked, the large-scale user privacy data would be leaked; (2) the service result is more normal, because only the user’s position factor is taken into consideration and the user social attributes such as personal preferences are ignored. Compared with a centralized location service system, the centralized location data server is not used in the distributed location service system. This avoids the risk of the server become a communication bottleneck and attack point, but it is still necessary to consider data privacy protection among neighboring users. Therefore, how to ensure the privacy of the user location and provide high quality personalized location services in the distributed location service system are the focus of our work. The location service architecture faces serious privacy threats caused by the disclosure of user position information. This can lead to link attacks on the relevant information. At present, lots of results have been obtained in the research on privacy protection for location services. These works aim to ensure that users could enjoy the location service and make attackers unable to obtain accurate position information. The main methods include K-anonymous methods, differential privacy protection methods, path obfuscation and random encryption [9]. As a traditional method, the K-anonymity method uses a position set to replace the user position, and the set contains the real user position and other fake K-1 positions. The K-top and K-mean clustering methods were universally adopted in K-anonymous privacy protection [10,11]. The density-based clustering method was proposed for the first time in [12]. In the background of this paper, the location density is of great significance. The high density of positions in a particular area means the user range of activities, and it can provide a positive effect in the service of recommending a location. Therefore, this paper used a selection method based on density prioritization to establish neighborhood groups.

Aside from the privacy protection methods based on location data, there are some methods based on LBS system structures [13,14,15]. For example, [13] cached part of the data on the mobile phone with the aid of caching ideas in distributed systems. In this method, the local cache could be queried so that the location information would not be exposed to the server. Reference [14] used the combination method of caching and user collaboration to prevent users from sending requests directly to the server as much as possible. Each user has a cached reservation for recent request results. Reference [15] used a P2P structure to protect location privacy and reduce the possibility of position privacy leaks through the capabilities of mobile devices and collaborative recommendations. The focus of the abovementioned researches was reducing the risk of privacy leakage in distributed systems and K-anonymity and caching mechanisms were adopted, but the location service recommendation method and quality problems were ignored. In brief, only the geographic location factor of the user is considered and the recommendation method in the location service is simple. For example, all the users with the same geographic location would get the same location service result.

Collaborative filtering recommendation algorithms have been widely used in the e-commerce field. The similarity of consumer behavior is used to realize personalized recommendations of goods. More detailed information leads to more accurate recommended results, but this algorithm is seldom used in location services. In order to improve the distributed location service, the collaborative filtering recommendation method was used to recommend location services for users, and two different location service recommendation methods based on user position profile and user position were designed. When the service requester is in a familiar area, the collaborative filtering recommendation method based on the user position profile similarity measure is adopted. The similarity of location nodes among users could be fully compared resulting in higher quality location service results in this method. When the service requester is in a relatively unfamiliar location area, the collaborative filtering recommendation method based on the user position similarity measure is adopted. This method focuses on the user’s current real-time position similarity measure, and the calculation amount is relatively small, so service results could be quickly achieved but the high quality of service could not be guaranteed. In the process of using collaborative filtering recommendation algorithms, users also face the risk of data information privacy disclosure. Therefore, there are some privacy protection methods for collaborative filtering recommendation algorithms, including the random interference method and group anonymous method [16,17,18,19]. Reference [8] based on the distributed system used simple location area overlap to recommend location services with only the geographic location results, so a high quality of service could not be guaranteed. Therefore, this paper proposes a collaborative filtering location service recommendation algorithm which is suitable for location services, where the position profile for each user was established based on the distributed structure, a new location scoring method was designed and the data was preprocessed based on density clustering. The user position profile was more clustered and more user social attributes could be described in our work. Finally the service to the LBS service requester was provided by using the profile information of the neighboring users. In contrast with the scheme proposed in [8], the proposed scheme fully considers the social attributes among the members of adjacent groups, although a peer-to-peer system structure was also adopted in this paper. Two collaborative filtering recommendation methods based on user position profile similarity and user position similarity were designed to recommend location services. Besides, homomorphic encryption was used to encrypt the location data to ensure the location privacy in the data transmission process. The proposed scheme in our work could provide higher quality location services and stricter privacy guarantees.

## 3. Methods

### 3.1. Description of the Scheme

A new collaborative filtering recommendation location service (CFRLS) method under distributed system conditions is proposed in our work. In this method, each user constructs their respective position profiles based on density metrics. When a user needs a location recommendation service, his own position information and service request are sent to the nearest neighbor, and then the neighbor users meeting the density measurement requirements adopt the collaborative filtering algorithm recommendation to provide LBS service results. Finally, the user performs secondary screening on the LBS service results recommended by the neighbor users to obtain satisfactory location services. If no satisfactory recommendation is obtained, the neighborhood user centroid position information is used to construct the K-anonymous dataset for sending service requests to a server.

By taking into account the user’s social attributes, a density clustering algorithm was adopted to construct a neighbor group. In this density clustering algorithm, a higher density indicates that the user is more familiar with the area, resulting in higher service quality. It should be noted that the collaborative recommendation method used in this paper is different from the traditional collaborative filtering algorithm. The traditional collaborative filtering algorithm is a more popular product recommendation technology. This traditional algorithm recommends products to users using historical data, machine learning and other technologies [20], but the group profile data were used to recommend and homomorphic encryption algorithm was used to transfer location data in the proposed method. Besides, this method used distributed systems to balance the computational load problem. Based on the distributed system structure, two different collaborative filtering recommendation algorithms were designed for user position profile and user position information to recommend LBS services in this strategy. It overcomes the communication bottleneck and focused attack of centralized anonymous system structures and improves the quality of the location service results.

### 3.2. Profile Construction

Based on our previous work in [8], a new location information profile construction method was designed in this paper. Each user collects its own position information to form position profile, which is composed of the node position, the topic tag and the score. In forming position profile, the density of the position relative position contour was prioritized. Besides, the location dwell time and the frequency of visits generated a score were considered. The description of the user location information profile was shown in Equation (1):

(1) $$\{\begin{array}{l} A=\left\{\left(L_{1},l_{1},s_{1}\right),\left(L_{2},l_{2},s_{2}\right),\left(L_{3},l_{3},s_{3}\right)\ldots \ldots \left(L_{k},l_{k},s_{k}\right)\right\}\\ D=\sum_{1\to k}\left(1+s_{k}\right)/A_{area} \end{array}$$

Each user node is represented by the user’s position profile *A*. Suppose the position profile is a collection of *k* users position information, and the element in the collection is a (*L*(*x, y*), *l*, *s*), where *L*(*x*, *y*) is the position of the user, (*x*, *y*) is the position coordinate, and *l* is the subject label of *L*(*x*, *y*) position of, *s* is the score. *D* is the absolute density of the user position profile, which is expressed as the number and score of the position points in the user position profile and the value of *D* is directly proportional to the user’s familiarity with the area. Besides, *s_k_* is the ratio of the position profile area and *s* represents the user’s score at *L*(*x*, *y*). Scoring function design was shown in Equation (2):

(2) $$\{\begin{array}{lll} \gamma =\alpha t+\beta c & & \left(\alpha >0,\beta >0,\alpha +\beta =1\right)\\ S\left(\gamma \right)=\frac{\gamma }{a+b\gamma } & & \left(a>0,b>0\right) \end{array}$$

In Equation (2), *t* represents the residence time in the *L*(*x*, *y*) position, *c* represents the times the user accesses in the *L*(*x*, *y*) position, (*α*, *β*) represent the proportion of the residence time and the number of visits in the score result, respectively. The *S*(*γ*) represents the users score at *L*(*x*, *y*), *a* and *b* are constant greater than zero, because of $S^{\prime }\left(\gamma \right)=\frac{a}{{\left(a+b\gamma \right)}^{2}}>0$, so the function $S\left(\gamma \right)=\frac{\gamma }{a+b\gamma }$ monotonically increases in [0,+∞) and the range is [0,1/b). The *b* value is used to control the upper limit of the score. In addition, the a value is used to control the rate of scoring growth, and because of $S^{\prime }\left(0\right)=\frac{1}{a}$, it means the larger the value of a causes the slower the growth of the service score. In our work, *a* = 0.8, *b* = 0.2 were set, so the location score range was [0,5) in this paper.

The steps to construct a profile algorithm are as follows:

Input: empty set *A*; position *x*; coefficient *α*, *β*;

Output: position profile set *A*

1: for all *a*ϵ*A* do

2: if *a.L* == *x.L* // if the *x* position already exists

3:   {*a.t* = *a.t* + *x.t;*

4:    *a.c* = *a.c* + *x.c*;

5:    *a.s* = *S*(*a.γ*);} // update position score information of a

6: *x.s* = *S*(*x.γ*); // calculate position score of *x*

7: insert *x* to *A*;

8: return *A*

In order to improve the usability of the position information profile and the service quality, pretreatment was performed on the collected user position profile in this paper. First, individual positions with high privacy needs were deleted according to the user-defined; Second, the divorced points were deleted, which is individual position off the center in position profile. In addition, a small number of divorced points with a lower score deviate from the user’s position profile area resulted in greatly reducing the user’s profile density *D*, and thus these points were deleted in the position profile. In the construction of the position profile, density clustering algorithm [12] was used to determine the centroid position of the user’s position profile and identify and delete the divorce points. The local density parameters (*ρ_i_*) and the nearest neighbor distance parameter (*δ_i_*) of position were calculated according to Equations (3) and (4), respectively. *ρ_i_* indicates that the distance from node *i* is less than the number of *d_c_* nodes The Equations (3) and (4) were shown as follows:

(3) $$\rho_{i}=\sum_{j}\chi \left(d_{ij}-d_{c}\right)$$

where χ(x)=1, *x* < 0 and χ(x)=0, *x* > 0, *d_c_* was set as distance threshold:

(4) $$\delta_{i}=\underset{j:\rho_{j}>\rho_{i}}{\min}\left(d_{ij}\right)$$

Besides, the nearest neighbor distance parameter (*δ_i_*) for the special nodes with the highest local density was calculated in Equation (5):

(5) $$\delta_{i}=\underset{j}{\max}\left(d_{ij}\right)$$

The position with bigger *ρ_i_* and *δ_i_* is regarded as the center of the position profile. The position with smaller *ρ_i_* and bigger *δ_i_* is regarded as outlier.

The preprocessing steps of position information profile are as follows:

Input: set *A*; distance threshold *d_c_*;

Output: position profile set *A*; position profile absolute density *D*; centroid position *key*;

1: for all *a*ϵ*A* do

2:  {calculate *a*.*ρ*

3:   calculate *a.δ*}

4: for all *a*ϵ*A* do

5:  if min(*a*.*ρ*)&&max(*a.δ*) // if *a* as outlier

6:   delete *a*

7:  if max(*a*.*ρ*)&&max(*a.δ*) // if *a* as the centroid position

8:    *A*. key = *a*;

9: calculate *A.D*

10: return *A*, *D*, *key*

The user’s original position information profile was constructed according to the above two steps, where *A* is a position set and a candidate set of recommended location services; *D* is the absolute density of the profile as a factor to measure the familiarity of the user in the profile area; *key* is the centroid position of the user profile area. A K-anonymous data set was constructed when the recommended location service failed. In order to prevent privacy risks among neighboring users, generalization was performed on original position profile result in a rectangular area *A_area_* that covers all position nodes to represent the user position information profile. At the same time, random offset processing was performed on centroid position in construction for preventing centroid position disclosure [8].

### 3.3. LBS Service Process

In the scenario designed in this paper, the user Alice is supposed to need to request LBS service at location *L*, so Alice broadcasts the service request information to neighboring users. The location service request information was sent according to different situations. In the first situation, the position profile and service request were sent when the position profile contains its own position. In the second situation, location generalization area and service request content were sent when the position profile does not contain its own position. For the above two different situations, two collaborative filtering recommendation location service methods were designed respectively. The first one is a recommendation method based on the position profile recommend a higher location service according to the similarity of the position information profile; the second one is the recommendation method based on the position.

When the neighboring user Bob receives Alice’s request, he firstly calculates the relative density *μ* of his own profile to Alice’s position *L*. If the density *μ* is greater than the preset density metric threshold, the condition is satisfied. Then Bob recommends the service according to the location profile or the user location and sends the recommendation result. If the density *μ* is not satisfied, the service request is not responded. Relative density measurement formula was shown in Equation (6):

(6) $$\mu =\sum_{i}\chi \left(d_{ij}-d_{c}\right)\;i=\left\{1,2,3\ldots \ldots k\right\}$$

where χ(x)=1, *x* < 0 and χ(x)=0, *x* > 0, *d_c_* is the measure of the position distance, a small *d_c_* value could be set in a concentrated user area to filter high quality neighboring users, whereas a larger *d_c_* value could be set in a sparse user area to ensure sufficient number of neighboring users. The *d_ij_* is the distance from the point *L_i_* to the point *L*, the relative density *μ* corresponds to the distance to the point *L_j_* is less than *d_c_*. The larger the relative density *μ* means the neighboring user Bob profile is more concentrated on the position *L* resulting in higher service results.

When the LBS service is requested, the position information would be sent to the neighboring nodes. However, there is the certain risk of position information being leaked when the position information data is directly broadcasted to the neighboring nodes. Therefore, Paillier encryption was used to encrypt position information in this paper [21].

When user Alice requests LBS service, the position *L* and service request content *label* would be sent. The algorithm was generated using private key and the algorithm was used to generate a pair of public key and private key (*pk*, *sk*) for the encryption scheme (*E*, *D*, *K*), then the request information, *R* = (*pk*, *E*(*pk*, *L’*), *Q*(*label*)) was sent to the neighboring user Bob, where *pk* is the public key generated by running key construction algorithm, *E*(*pk*, *L*) is the identifier of the encrypted position information; *Q*(*label*) is the queried content.

**Figure 3:**
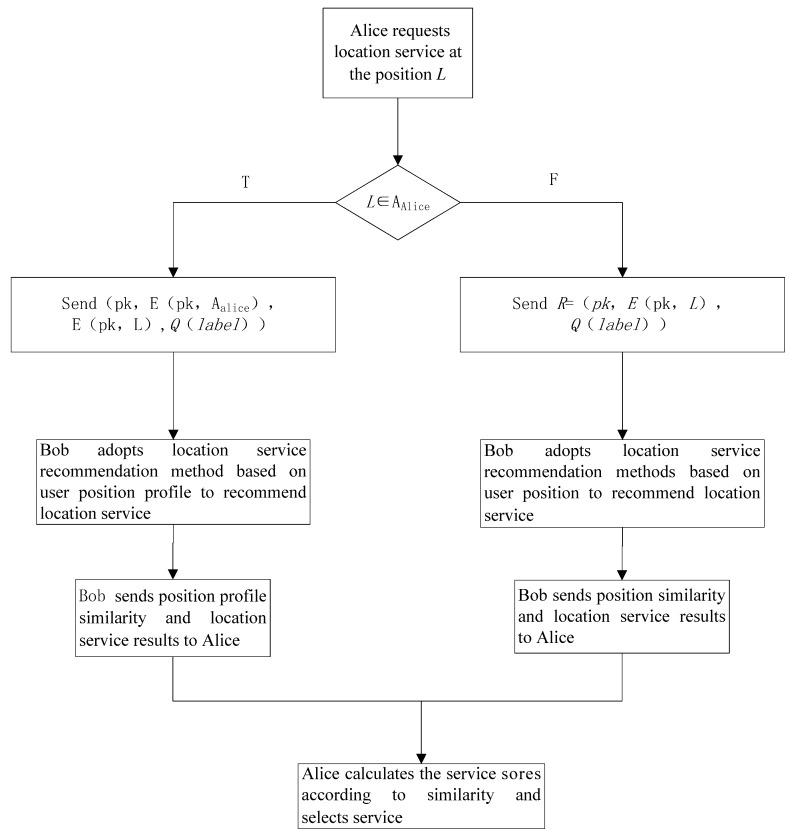
LBS service flow chart.

When the neighboring user Bob receives the requested information from user Alice, his position *L_b_* would be performed using encryption operation *E*(*pk*, *L_b_*) to obtain the encrypted position *L_b_’*. The result of the position profile encrypted using *E*(*pk*, *L’*) of the user *A* is supposed as *L_a_’*. According to the characteristics of the Paillier encryption method, the *L_a_’* and *L_b_’* were operated using the algorithm designed in this paper and the calculation results were not affected. At the same time, it also avoids the risk of privacy leakage caused by plaintext calculations and also increases the computational burden. The request LBS service flow chart is shown in Figure 3.

#### 3.3.1. The Recommended Method Based on the Position Profile

The recommendation method based on the position profile is adopted by the neighboring users when they receive the position profile of requesters. The higher similarity of two location information causes the quality of the service to be higher. According to the characteristics of this strategy, the Euclidean similarity measure method was used as a similarity measure method. *k* common position points were supposed in two position profiles, where the scores of the *L_i_* position are *x_i_* and *y_i_*, respectively. Then the Euclidean distance between the two position profiles is given by Equation (7):

(7) $$d\left(x,y\right)=\sqrt{\left(\sum^{\text{​}}{\left(x_{i}-y_{i}\right)}^{2}\right)}$$

The similarity between the two position profiles is shown in Equation (8):

(8) $$\mathrm{sim}\left(x,y\right)=\frac{1}{1+d\left(x,y\right)}$$

Then, the position profile similarity and the recommended location service set are returned to the requester. If the set contains a total of *n* location service results, any location *L_i_* and *m* recommended users are supposed. Then there exists an attribute set (*s_j_*, *sim_j_*) for *l_i_*, where *j* = 1, 2, 3 … *m*, *s_j_* is the score of *L_i_* for the user *A_j_*, *sim_j_* is the similarity between the user *A_j_* and the service requester *B*, and the location service prediction score of the *L_i_* from user *B* were calculated according to Equation (9):

(9) $$s_{i}=\frac{\sum^{\text{​}}s_{j}sim_{j}}{\sum^{\text{​}}sim_{j}}\;\left(i=1,2,3\ldots \ldots n;j=1,2,3\ldots \ldots m\right)$$

**Figure 4:**
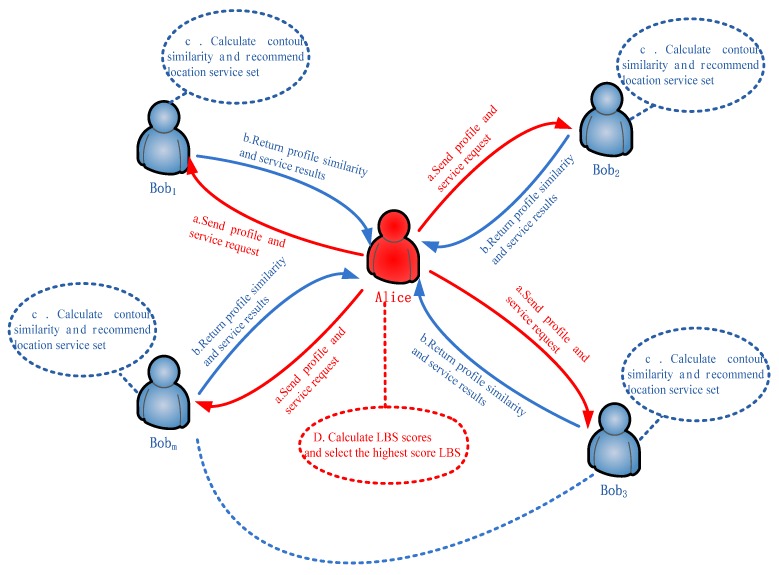
Collaborative filtering recommendation algorithm based on position profile.

Finally, the location service with the highest prediction score was used as the final location recommendation result. The schematic flowchart of the collaborative filtering recommendation algorithm based on position profile is shown in Figure 4.

#### 3.3.2. The Recommendation Method Based on Requested Position Information

The recommendation method based on the position information is adopted by the neighboring users when they receive the temporary position information set from user *A*. The degree of matching between the position profile and the received position information is calculated, and the higher matching degree causes a higher quality of the recommended location service. In other words, the position profile contains the request position and the recommended position, the possibility of successful recommended result is greater. According to the description, the similarity was called for the match degree, the *k* temporary positions were supposed, the similarity was calculated using Tanimoto coefficient, and the equation was shown in Equation (10):

(10) $$\mathrm{sim}=\frac{N_{c}}{N_{a}+N_{b}-N_{c}}$$

where *N_a_* is the number of position nodes in the temporary position information set of user *A*, *N_b_* is the number of position nodes in the position profile of user *B*, and *N_c_* is the number of common position nodes in two position information, sim is the similarity. Then, the similarity and the recommended location service set were returned to the location service requester. In addition, the method of predicting the score for the position node is the same as that based on the position profile.

## 4. Security and Characterization

A theoretical analysis of the data security and program effectiveness were provided for the proposed strategy. For the privacy protection features of the strategy, the advantages of the proposed privacy protection were illustrated by comparing the characteristics with the solutions proposed in P2P [6] and MobiCrowd [7]. First of all, most of the existing privacy protection methods are based on a centralized system structure. When the service requester sends a service request to the server, its own position information is easily leaked. Therefore, the program in P2P provided LBS service based on a distributed system structure, and K-anonymity was constructed to send position information using the position of neighbor nodes. This scheme could provide a better K-anonymity data set, but it did not consider the privacy and security of the position information among the neighboring nodes. In addition, the number of access servers is not reduced and the risk of privacy disclosure still exists in this strategy. The distributed system structure was used in the MobiCrowd strategy. The history request server results were shared among the neighboring nodes and trying to reduce the number of access server requests to reduce the risk of privacy leakage, and the cold-start problem still exists, but the privacy threat from the server and the privacy protection of the neighboring users were neglected.

Compared with the strategy in P2P, the individual privacy problems of users were considered in the process of constructing user position profile. The positions with higher privacy requirements and the divorce points with special meaning were deleted using a density clustering algorithm. In addition, the position information was generalized and replaced by a rectangular area with settable increments and the rectangular area was randomly offset in order to prevent the center attack. The same approach was used in recommending service information for the nearest user. Thus, the position information of the requesting service user was protected effectively.

At the same time, the encryption algorithm based on Paillier encryption algorithm was used to transmit data between neighbor nodes. In the designed system, the encrypted rectangular area data was only operated by neighboring nodes to ensure the position information privacy of service requester. In order to solve the large amount of computation in encryption, the algorithm proposed in this paper simplified the matching algorithm of neighboring users as much as possible. Furthermore, each node only need to encrypt its own position profile in service request response process based on the distributed structure, which result in effectively reducing the calculation of a single node. In addition, the K-anonymous data set was constructed using neighbor positions, the centroid position was also offset to reduce the risk of real position leakage and effectively prevent collusion attacks.

Compared with MobiCrowd, the position profile was used to recommend location services in our strategy, but the location server did not need to visit to construct the position profile. Position information was stored in its own memory, thus there is no privacy leak in the proposed process.

## 5. Results and Discussion

The CRAWDAD dataset was used in experiment, which includes the movement track data of 536 taxis in San Francisco within one month [22,23]. This dataset is widely used in the analysis and research of position data. In experiment, the characteristics were verified and analyzed for the algorithm, LBS service request, responses and three stages of collaborative filtering recommendation location service algorithm. The evaluation indicators mainly include the position data outline characteristics, the number of response users, the number of service results and the probability of successful service recommendation. The data availability, the provision of location service quality of the proposed strategy are verified and compared with DCRLS [8]. The system architecture, algorithm efficiency and communication costs were analyzed and compared with the algorithms MobiCrowd and DCRLS.

### 5.1. Analysis and Verification of Algorithm

In the strategy proposed in this paper, the position data points with longer residence time were collected according to the density prioritization, and then the position points in the position profile were scored. When there is an LBS service request, the similarity of the neighboring users is calculated, and the score of the recommended location service is predicted according to the similarity, and the final location service is determined according to the score. Because the position information set contained in the node position profile has an important influence on the quality of the LBS service recommendation result, the relevant characteristics of the node data profile were verified and analyzed on the real dataset. In the analysis and verification process, the number of nodes of the position profile, some factors including the density measure of the position profile and the score of the node were specific analyzed. Besides, these analysis and verification were compared with DCRLS.

The real dataset contains the position information of 536 taxis for a month, which is represented by their latitude and longitude data, and timestamps corresponding to the position point. The algorithm proposed in this paper processes the position information of each taxi object to construct the corresponding position profile. In the experiment, the position information of the object is constructed according to the residence time parameter of the node, and then the local density parameter and the distance parameter of each neighborhood are calculated. At the same time, the two parameters are used to determine the location of the centroid and remove the divorced point. Then the location node in the position profile is determined and rated. In order to find out where the object resides, the average moving velocity (*v*) between two adjacent timestamps of an object is calculated. Then the *v* is used to measure the object’s dwell time in the corresponding area. Obviously when *v* = 0, it indicates that the object is still in this time period. Considering the special nature of objects as taxis, stationary position is only considered to form corresponding position information. In the experiment, the position information of 536 taxi objects was processed using the profile construction algorithm, and then the corresponding position information profile constructed. The statistics and analysis were conducted for data. The *α* = 0.5, *β* = 0.5, *a* = 0.8, *b* = 0.2, *d_c_* = 100 and density threshold *μ*’ = 200. First of all, we selected six taxis from 536 taxi objects for instance analysis, and finally gave the data of all the objects.

**Figure 5:**
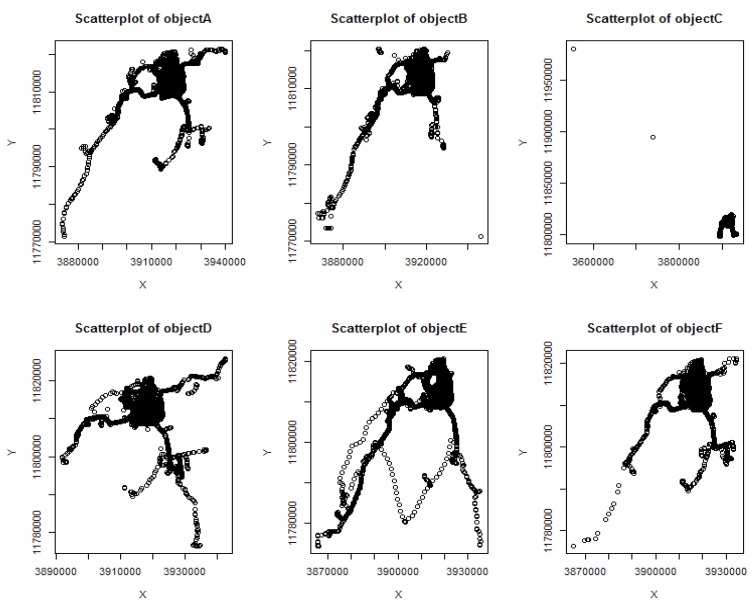
The scatter plot of object original locations.

**Figure 6:**
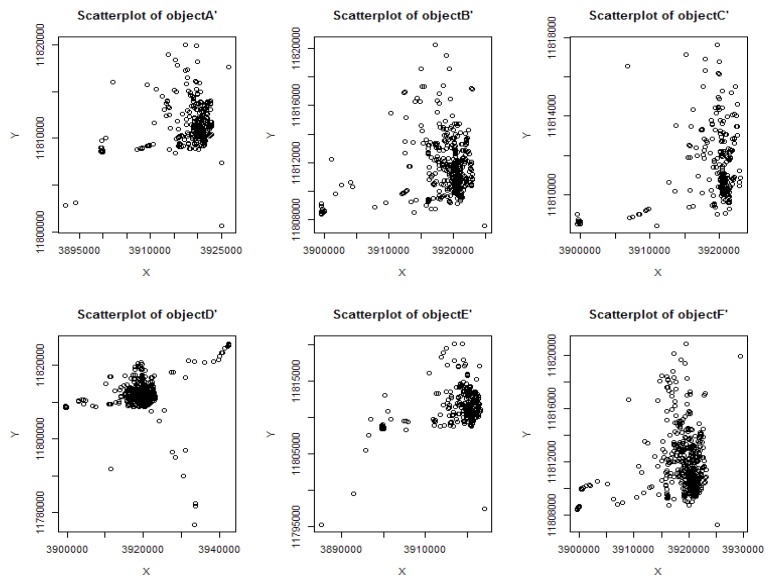
The scatter plot of object position profiles in DCRLS.

**Figure 7:**
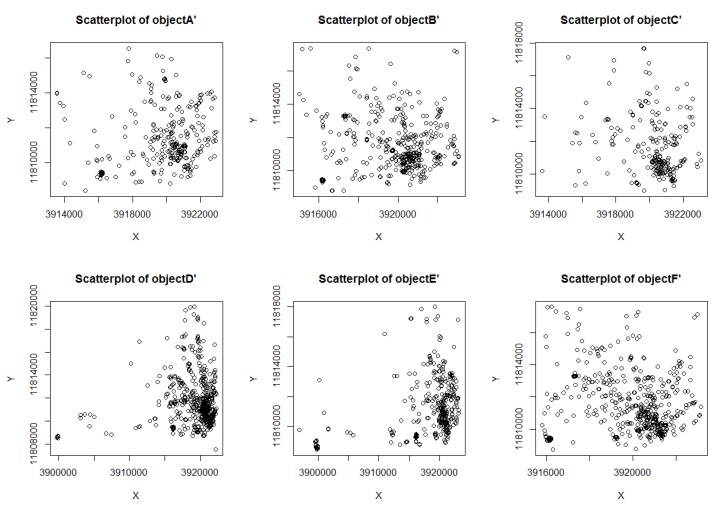
The scatter plot of object position profiles in CFRLS.

The six taxi objects were randomly selected as *A*~*F*, their original position data scatter shown in Figure 5. The corresponding scatter plot of the position profile construction algorithm DCRLS was shown in Figure 6. The corresponding scatter plot of the position profile construction algorithm proposed CFRLS was shown in Figure 7.

From Figure 6 and Figure 7, it can be intuitively seen that the algorithm proposed in this paper shows better aggregation and higher density. The possible reason is removing these divorced points based on density measure in constructing position profiles. In order to further analyze the availability of the proposed algorithm, the data of the six moving objects were counted, including the original rectangular area and the number of the position, the rectangular area of the position profile and the number of the position, the ratio of the original area to the profile area and the number ratio between the original position and the profile position data. These data are shown in Table 1.

**Figure 8:**
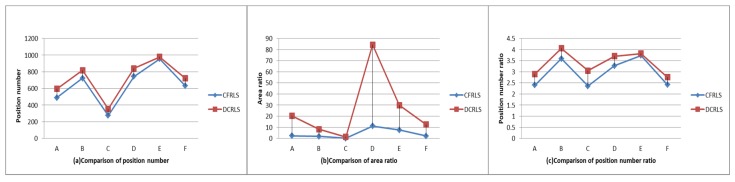
Comparison of position profile data.

From Table 1, it can be seen that the ratio of the number of construction position/the original position ratio (ratio NP) was about 3%. It showed that about 3% of the original set of objects was used to form the position profile in the proposed profile construction algorithm. The node position profile area ratio (ratio RE) is related to the density of the moving object’s active area. The area ratio of the position profile (ratio RE) constructed in this paper is less than the value of DCRLS. The possible reason is removing the divorce points. According to the scatter plot of data and node position information in Table 1, it was obvious that outliers with shorter dwell time and fewer accesses would be removed using the proposed algorithm, which result in higher quality utilization. In addition, there are less location data in the constructed profile caused improvement of data operation and storage efficiency. Compared with DCRLS, correlation data of position profile were shown in Figure 8.

**Figure 9:**
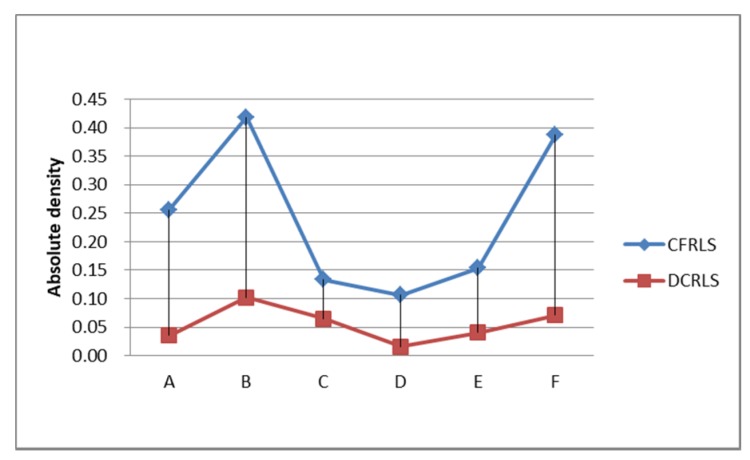
Comparison of absolute density.

The absolute densities of the information profile in two algorithms were shown in Figure 9. From Figure 9, it can be clearly seen that the number of position points and the ratio and area in CFRLS are lower than them in DCRLS, but the absolute density (abdensity) in CFRLS is higher than DCRLS. In other words, despite the lack of location information, but the data quality has greatly improved.

The position profile of all the moving objects in the data set was constructed using proposed algorithm in the experiment. There are 536 moving objects, 11,219,955 position points and an average of 20,930 position points in the original dataset. There are 312,827 points and an average of 601 points in constructed position profile. The average ratio of position points is 2.8%. The above data shows that the proposed position profile construction algorithm could effectively constructs the position information profile of each mobile object and the constructed position information profile has higher data availability.

### 5.2. Algorithm Analysis and Verification of Service Request and Response Process

Experimental simulation and analysis were performed using LBS service request and response process algorithm in the proposed strategy. The parameter setting is the same as the previous section. 100 users were randomly selected as LBS service requesters in data set. In order to facilitate the description, the service request content theme reduced to “the next hour where I will go?” combining data set characteristics. It should be noted that in practical applications, service themes and related parameters can be flexibly set according to the actual situation.

**Figure 10:**
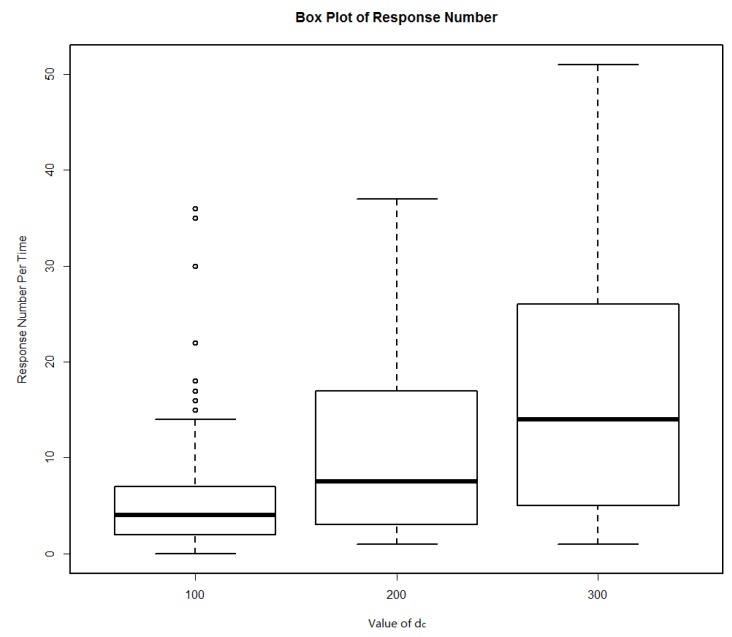
The number of response users.

According to the algorithm proposed in this paper, the LBS service request and response process were as follows: when the user made the LBS service request, a distance truncation parameter was set as *d_c_*, the service requests were sent; moving objects less than 100 m from the user were selected in 5 min, then the objects determined the density metric based on the truncation parameter *d_c_* and recommended location service using collaborative filtering method. When the parameter *d_c_* was set to 100, 200 and 300 m for three groups of experiments, 100 service requests were randomly generated in each group and the number of response user box diagram was shown in Figure 10.

When the *d_c_* values were 100, 200, 300 m, respectively, the specific number of the corresponding response users was shown in Table 2.

**Figure 11:**
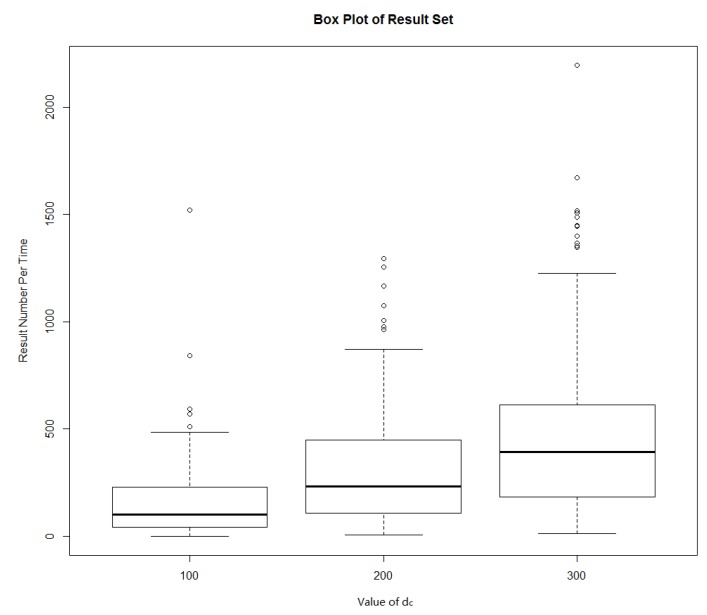
The number of service results.

At each service request, position information in the own position profiles of service responding users that matches the topic requirements were recommended to service requestors. Then, service requestors would obtain a location service alternative collection. In the experiment, we counted the number of service candidate set elements obtained from each service request in three experiments when the *r* values were respectively 100, 200 and 300 m, and the results are shown in Figure 11. When the *d_c_* values were 100, 200, 300, respectively, the specific numbers of the service results are shown in Table 3. From Figure 11 and Table 3, it can be clearly seen that the number of response users increases as the value of *d_c_* increases, which resulted in an increase of service recommendations. In the data set, there is no user response when the *d_c_* value was 100; the number of response users and recommended service results was quite satisfactory when the value of *d_c_* was 200 and 300.

Due to the geographical randomness of service requesters, it is inevitable that there are fewer users responding to the request and the higher-quality location service recommendation result may not be obtained. Therefore, the K-anonymity [6] was used in the proposed scheme as a supplementary solution in this case.

### 5.3. Comparison among CFRLS, DCRLS and MobiCrowd

The distributed system structure was adopted in the proposed algorithm CFRLS, DCRLS and MobiCrowd. The differences among these three algorithms lie in the system architecture, the dependency degree on the server, the execution efficiency and communications cost. The CRAWDAD data set was used in experiment analysis for comparison. The comparison in architecture of the system was shown in Table 4. The MobiCrowd algorithm has high dependence on the server. The reason is the access frequency to the location server would reduce with position information caching. Compared with MobiCrowd algorithms, it is noteworthy that the server would be accessed only if the location service recommendation fails in DCRLS and CFRLS algorithms. The two proposed algorithms have lowest dependence on the server.

**Figure 12:**
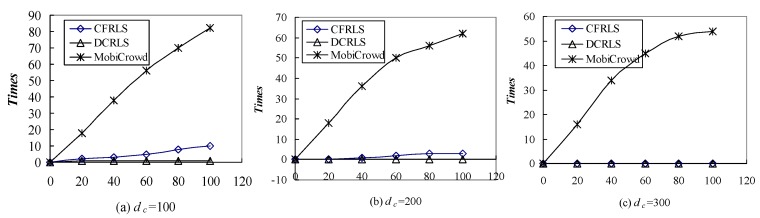
Comparison of the access server number with different algorithms.

In order to further compare the number of the location server access in three algorithms under the same conditions, the parameters *d_c_* was set as 100, 200, 300 m, the parameter *k* of K-anonymous was 10. When the 100 location services were requested randomly in three algorithms, the comparisons of location server access were shown in Figure 12.

From Figure 12, it can be clearly seen that there are significant differences among the three algorithms in accessing the location server. In the initial stage of the service, the location serve was accessed due to cold-start problems in MobiCrowd algorithm. After the accumulation of location service information, the dependence on the location server was gradually reduced in MobiCrowd. In contrast, the dependence on the location server was lower in proposed DCRLS and CFRLS algorithms. In the CFRLS algorithm, the lower *d_c_* value would cause lower service recommendation number and higher access server times. Thus, suitable setting of density threshold according to different application environment could reduce dependence on location server and the privacy risk posed by third-party untrusted location servers.

**Figure 13:**
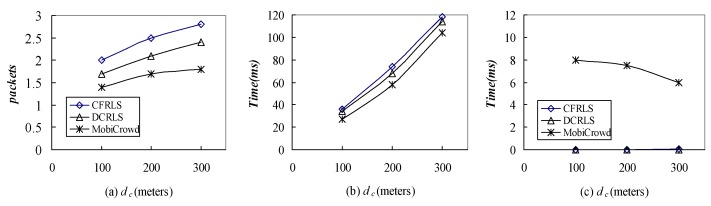
Comparison of (**a**) communication costs, (**b**) client costs, (**c**) server costs with different algorithms.

The average costs of three algorithms under the same conditions were compared and the number of TCP/IP packets was used as metrics. Besides, the average time costs of server and client were compared in our experiment. The results were shown in Figure 13.

From Figure 13a, it can be seen that the average communication load of CFRLS strategy was higher than DCRLS and MobiCrowd algorithms. The possible reason is that the data communication load in this strategy mainly focuses on the communication among the neighboring nodes. The MobiCrowd algorithm had lower communication load due to its caching mechanism. From Figure 13b,c, the time spent on the client and the server was compared for the three algorithms. In MobiCrowd algorithm, the server-side time costs gradually reduced with the cumulative increased due to the probability of sharing location service information between nodes increased. From the analysis results, CFRLS algorithm spent more on client time compared with DCRLS and MobiCrowd algorithms. It is due to user client profile density measurement and collaborative filtering recommendations for location information, but the quality of location services was improved. In addition, the server-side time spent almost could be ignored. This also precisely verifies the original design of the algorithm in this paper, the communication spending load was scattered in the neighboring nodes to reduce the privacy leak to visit the location server.

## 6. Conclusions

In the present work, a collaborative filtering recommendation location service strategy based on the density measure with considering the social attributes of users was proposed to solve the problem of lower quality service in existing distributed structure location service systems. Two filter algorithms based on position profiles and position points were designed for recommendation, respectively. The theoretical and simulation results were analyzed using real datasets for the algorithm proposed in this strategy. The analysis showed that the strategy proposed in this paper could provide a sufficient number of service response users and higher quality service result sets for reducing the frequency of user access to the LBS server, overcoming the communication bottleneck and focused attacks in the centralized anonymous system structure. The proposed strategy ensured the privacy of user position information security. The proposed strategy might be considered as a good alternative to protect privacy in the future. The timing problems of user position points in user profile construction will be considered to further improve the recommendation quality of LBS services.

## Figures and Tables

**Table 1:** Object profile data.

Name	Number OP	Number P	Ratio RE	Ratio NP	Abdensity
object A	20,543	489	2.3%	2.4%	0.26
object B	20,159	724	1.79%	3.59%	0.42
object C	11,616	274	0.12%	2.36%	0.13
object D	22,694	743	11.11%	3.27%	0.11
object E	25,611	957	7.64%	3.74%	0.15
object F	26,165	632	2.09%	2.42%	0.39

**Table 2:** The number of response user.

*d_c_* (m)	Minimum	Maximum	Average	Failure Rate (%)
100	0	36	5.88	0.01
200	1	37	11.22	0
300	1	51	17.26	0

**Table 3:** Number of service result sets.

*d_c_* (m)	Minimum	Maximum	Average	Failure Rate (%)
100	0	1520	169.11	1%
200	5	1294	340.56	0%
300	13	2195	520.5	0%

**Table 4:** The comparison of architectures with different algorithms.

Item	CFRLS	DCRLS	MobiCrowd
Architecture tiers	2 tiers	2 tiers	3 tiers
Dependence on trusted third party	Low	heavy	medium
Privacy protect among peers	good	low	weak
